# Bis[*N*′-(3-cyano­benzyl­idene)isonicotino­hydrazide]silver(I) trifluoro­acetate

**DOI:** 10.1107/S1600536809029183

**Published:** 2009-08-08

**Authors:** Cao-Yuan Niu, Hai-Yan Zhang, Yu-Li Dang, Chun-Hong Kou

**Affiliations:** a College of Sciences, Henan Agricultural University, Zhengzhou 450002, People’s Republic of China

## Abstract

In the title compound, [Ag(C_14_H_10_N_4_O)_2_]CF_3_CO_2_, the Ag^I^ ion is coordinated by two N atoms of the pyridine rings of two *N*′-(3-cyano­benzyl­idene)isonicotinohydrazide ligands in a nearly linear geometry. In the crystal structure, a combination of close contacts formed *via* Ag⋯N inter­actions [Ag⋯N = 3.098 (2) and 3.261 (2) Å] from symmetry-related mol­ecules and inter­molecular N—H⋯O hydrogen bonds between CF_3_CO_2_^−^ anions and the hydrazone groups of two ligands give rise to chains. Furthermore, there are Ag⋯O inter­actions with a separation of 2.765 (2) Å between chains. The F atoms of the CF_3_CO_2_^−^ anion are disordered over two sites with refined occupancies of 0.593 (5) and 0.407 (5).

## Related literature

For related silver complexes, see: Dong *et al.* (2004 ▶); Niu *et al.* (2008 ▶, 2009 ▶); Sumby & Hardie (2005 ▶); Abu-Youssef *et al.* (2007 ▶); Zheng *et al.* (2003 ▶).
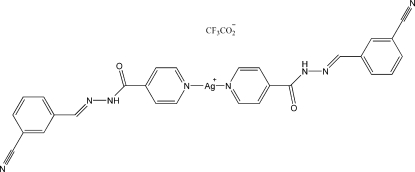


## Experimental

### 

#### Crystal data

[Ag(C_14_H_10_N_4_O)_2_]C_2_F_3_O_2_
*M* *_r_* = 721.41
Triclinic, 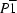

*a* = 7.5345 (14) Å
*b* = 13.744 (3) Å
*c* = 14.098 (3) Å
α = 86.562 (3)°
β = 88.126 (3)°
γ = 83.792 (3)°
*V* = 1448.2 (5) Å^3^
*Z* = 2
Mo *K*α radiation
μ = 0.77 mm^−1^
*T* = 173 K
0.32 × 0.22 × 0.17 mm

#### Data collection

Bruker APEXII CCD detector diffractometer
Absorption correction: multi-scan (*SADABS*; Sheldrick, 1996 ▶) *T*_min_ = 0.791, *T*_max_ = 0.881
8015 measured reflections
5306 independent reflections
4046 reflections with *I* > 2σ(*I*)
*R*_int_ = 0.025

#### Refinement

*R*[*F* ^2^ > 2σ(*F* ^2^)] = 0.046
*wR*(*F* ^2^) = 0.137
*S* = 1.03
5306 reflections
443 parameters
48 restraints
H-atom parameters constrained
Δρ_max_ = 0.95 e Å^−3^
Δρ_min_ = −0.83 e Å^−3^

### 

Data collection: *SMART* (Bruker, 2002 ▶); cell refinement: *SAINT* (Bruker, 2002 ▶); data reduction: *SAINT*; program(s) used to solve structure: *SHELXL97* (Sheldrick, 2008 ▶); program(s) used to refine structure: *SHELXL97* (Sheldrick, 2008 ▶); molecular graphics: *SHELXTL* (Sheldrick, 2008 ▶) and *DIAMOND* (Brandenburg, 2005 ▶); software used to prepare material for publication: *SHELXL97*.

## Supplementary Material

Crystal structure: contains datablocks I, global. DOI: 10.1107/S1600536809029183/lh2867sup1.cif

Structure factors: contains datablocks I. DOI: 10.1107/S1600536809029183/lh2867Isup2.hkl

Additional supplementary materials:  crystallographic information; 3D view; checkCIF report

## Figures and Tables

**Table:** 

Ag1—N5	2.143 (3)
Ag1—N1	2.147 (3)

**Table:** 

N5—Ag1—N1	174.20 (11)

**Table 2:** Hydrogen-bond geometry (Å, °)

*D*—H⋯*A*	*D*—H	H⋯*A*	*D*⋯*A*	*D*—H⋯*A*
N2—H40⋯O4^i^	0.88	1.93	2.805 (4)	172
N6—H39⋯O3^ii^	0.90	2.13	2.936 (4)	149

Symmetry codes: (i) 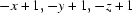; (ii) 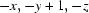.

**Fig. 1.**
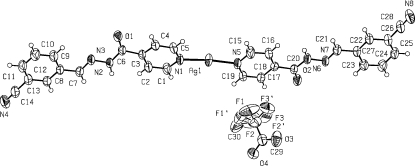
The asymmetric unit of (I) showing the atom-labelling scheme. Displacement ellipsoids are drawn at the 50% probability level. In the anion, the dashed lines indicate the minor component of disorder.

**Fig. 2.**
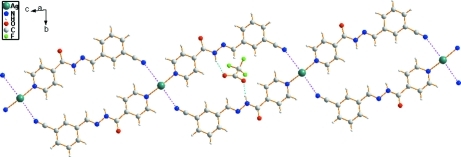
Part of the the one-dimensional chain formed via intermolecular hydrogen bonds indicated by green dashed lines and Ag···N interactions indicated by pink dashed lines.

## supplementary crystallographic information

### Comment

Silver coordination complexes with pyridyl organic ligands are of great interests for their utilities in fluorescent materials and antibiotic aspects (Dong *et al.*,  2004; Abu-Youssef, *et al.*, 2007). In the title compound, (I), the central Ag^I^ ion is coordinated by two nitrogen atoms from two pyridine rings of two different ligands, defining a slightly distorted linear coordination geometry (Fig. 1). Coordinating bond distances and angle around metal center are shown in Table 1. In the crystal structure, there are N—H···O hydrogen bonds between the hydrazone groups of 3-cyanobenzylidene isonicotinohydrazide ligands and CF_3_CO_2_^-^ anions (Table 2). In addition, there are weak Ag···N interactions between two neighbouring silver monomers with separations of 3.098 (2) and 3.261 (2) Å and Ag···O interactions between two neighbouring silver monomers with separations of 2.765 (2) Å. Hydrogen bonds and Ag···N interactions link parallel silver monomers together to construct one-dimensional chains (Fig. 2) and Ag···O interactions contribute to the three-dimensional structure.

### Experimental

A solution of AgCF_3_CO_2_ (0.022 g, 0.1 mmol) in CH_3_OH (10 ml) was carefully layered on a CH_3_OH/CHCl_3_ solution (5 ml/10 ml) of 3-Cyanobenzylidene isonicotinohydrazide (0.025 g, 0.1 mmol) in a straight glass tube. About ten days later, colourless single crystals suitable for X-ray analysis were obtained (yield about 43%).

### Refinement

C-bound H atoms were placed in calculated positions and refined using a riding model [C—H = 0.95 Å and *U*_iso_(H) = 1.2*U*_eq_(C)]. The N-bound H atoms were first introduced in calculated positions and refined freely with *U*_iso_(H) = 1.2*U*_eq_(carrier N). Three F atoms (F1—F3) of the trifluoroacetate anion are disordered over two positions, with maximum and minimum occupancies of 0.593 (5) and 0.407 (5), respectively. All C—F bond lengths were restrained to 1.26 (2) Å. Restraints of displacement parameters for three F or disordered F atoms were also performed. The final difference Fourier map had a highest peak at 0.96 Å from atom Ag1 and a deepest hole at 0.96 Å from atom Ag1, but were otherwise featureless.

### Crystal data

**Table:**

[Ag(C_14_H_10_N_4_O)_2_]C_2_F_3_O_2_	*Z* = 2
*M**_r_* = 721.41	*F*(000) = 724
Triclinic, *P*1	*D*_x_ = 1.654 Mg m^−^^3^
Hall symbol: -P 1	Mo *K*α radiation, λ = 0.71073 Å
*a* = 7.5345 (14) Å	Cell parameters from 2885 reflections
*b* = 13.744 (3) Å	θ = 2.1–25.5°
*c* = 14.098 (3) Å	µ = 0.77 mm^−^^1^
α = 86.562 (3)°	*T* = 173 K
β = 88.126 (3)°	Needle, yellow
γ = 83.792 (3)°	0.32 × 0.22 × 0.17 mm
*V* = 1448.2 (5) Å^3^	

### Data collection

**Table:**

Bruker APEXII CCD detector diffractometer	5306 independent reflections
Radiation source: fine-focus sealed tube	4046 reflections with *I* > 2σ(*I*)
graphite	*R*_int_ = 0.025
φ and ω scans	θ_max_ = 25.5°, θ_min_ = 2.1°
Absorption correction: multi-scan (*SADABS*; Sheldrick, 1996)	*h* = −9→9
*T*_min_ = 0.791, *T*_max_ = 0.881	*k* = −16→16
8015 measured reflections	*l* = −8→17

### Refinement

**Table:**

Refinement on *F*^2^	Primary atom site location: structure-invariant direct methods
Least-squares matrix: full	Secondary atom site location: difference Fourier map
*R*[*F*^2^ > 2σ(*F*^2^)] = 0.046	Hydrogen site location: inferred from neighbouring sites
*wR*(*F*^2^) = 0.137	H-atom parameters constrained
*S* = 1.03	*w* = 1/[σ^2^(*F*_o_^2^) + (0.0871*P*)^2^ + 0.05*P*] where *P* = (*F*_o_^2^ + 2*F*_c_^2^)/3
5306 reflections	(Δ/σ)_max_ = 0.001
443 parameters	Δρ_max_ = 0.95 e Å^−^^3^
48 restraints	Δρ_min_ = −0.83 e Å^−^^3^

### Special details

**Table:**

Geometry. All e.s.d.'s (except the e.s.d. in the dihedral angle between two l.s. planes) are estimated using the full covariance matrix. The cell e.s.d.'s are taken into account individually in the estimation of e.s.d.'s in distances, angles and torsion angles; correlations between e.s.d.'s in cell parameters are only used when they are defined by crystal symmetry. An approximate (isotropic) treatment of cell e.s.d.'s is used for estimating e.s.d.'s involving l.s. planes.
Refinement. Refinement of *F*^2^ against ALL reflections. The weighted *R*-factor *wR* and goodness of fit *S* are based on *F*^2^, conventional *R*-factors *R* are based on *F*, with *F* set to zero for negative *F*^2^. The threshold expression of *F*^2^ > σ(*F*^2^) is used only for calculating *R*-factors(gt) *etc*. and is not relevant to the choice of reflections for refinement. *R*-factors based on *F*^2^ are statistically about twice as large as those based on *F*, and *R*- factors based on ALL data will be even larger.

### Fractional atomic coordinates and isotropic or equivalent isotropic displacement parameters (Å^2^)

**Table:**

	*x*	*y*	*z*	*U*_iso_*/*U*_eq_	Occ. (<1)
Ag1	0.07174 (4)	0.80139 (2)	0.31966 (2)	0.07153 (17)	
N1	0.1905 (4)	0.8820 (2)	0.4222 (2)	0.0511 (7)	
N2	0.4458 (4)	1.0110 (2)	0.71224 (19)	0.0467 (6)	
H40	0.4975	0.9505	0.7165	0.056*	
N3	0.4965 (4)	1.0684 (2)	0.78081 (19)	0.0499 (7)	
N4	0.9740 (5)	0.9994 (3)	1.2069 (2)	0.0771 (10)	
N5	−0.0205 (4)	0.7130 (2)	0.2151 (2)	0.0542 (7)	
N6	−0.3086 (4)	0.5740 (2)	−0.06091 (19)	0.0499 (7)	
H39	−0.3230	0.6397	−0.0717	0.060*	
N7	−0.3701 (4)	0.5149 (2)	−0.12433 (19)	0.0473 (6)	
N8	−0.8578 (6)	0.5997 (3)	−0.5468 (3)	0.0829 (11)	
O1	0.2840 (4)	1.14121 (17)	0.63867 (18)	0.0630 (7)	
O2	−0.2138 (4)	0.44400 (17)	0.03838 (17)	0.0601 (6)	
O3	0.2518 (5)	0.2337 (2)	0.1519 (2)	0.0957 (11)	
O4	0.3698 (5)	0.1775 (2)	0.2890 (2)	0.0950 (11)	
C1	0.2540 (6)	0.8390 (3)	0.5037 (3)	0.0623 (10)	
H28	0.2629	0.7695	0.5111	0.075*	
C2	0.3069 (5)	0.8889 (3)	0.5766 (3)	0.0577 (9)	
H29	0.3509	0.8547	0.6331	0.069*	
C3	0.2955 (4)	0.9898 (2)	0.5673 (2)	0.0413 (7)	
C4	0.2324 (4)	1.0347 (2)	0.4829 (2)	0.0480 (8)	
H30	0.2240	1.1040	0.4733	0.058*	
C5	0.1822 (4)	0.9791 (3)	0.4135 (2)	0.0492 (8)	
H31	0.1392	1.0115	0.3560	0.059*	
C6	0.3404 (4)	1.0549 (2)	0.6427 (2)	0.0470 (8)	
C7	0.5898 (4)	1.0245 (3)	0.8475 (2)	0.0488 (8)	
H32	0.6173	0.9553	0.8495	0.059*	
C8	0.6539 (4)	1.0815 (2)	0.9204 (2)	0.0461 (7)	
C9	0.6270 (5)	1.1842 (3)	0.9143 (3)	0.0606 (10)	
H33	0.5639	1.2171	0.8623	0.073*	
C10	0.6902 (6)	1.2380 (3)	0.9820 (3)	0.0748 (12)	
H34	0.6709	1.3075	0.9768	0.090*	
C11	0.7812 (6)	1.1916 (3)	1.0576 (3)	0.0672 (11)	
H35	0.8269	1.2289	1.1040	0.081*	
C12	0.8062 (5)	1.0904 (3)	1.0659 (3)	0.0545 (9)	
C13	0.7428 (5)	1.0354 (3)	0.9974 (2)	0.0481 (8)	
H36	0.7606	0.9658	1.0037	0.058*	
C14	0.9005 (5)	1.0402 (3)	1.1449 (3)	0.0603 (9)	
C15	−0.1106 (6)	0.7536 (3)	0.1400 (3)	0.0700 (11)	
H22	−0.1284	0.8231	0.1327	0.084*	
C16	−0.1786 (5)	0.7013 (3)	0.0732 (3)	0.0590 (9)	
H21	−0.2389	0.7340	0.0203	0.071*	
C17	−0.1585 (4)	0.6002 (2)	0.0834 (2)	0.0417 (7)	
C18	−0.0668 (5)	0.5584 (2)	0.1616 (2)	0.0499 (8)	
H20	−0.0499	0.4890	0.1715	0.060*	
C19	−0.0005 (5)	0.6156 (3)	0.2244 (2)	0.0530 (8)	
H19	0.0627	0.5847	0.2772	0.064*	
C20	−0.2274 (4)	0.5316 (2)	0.0187 (2)	0.0442 (7)	
C21	−0.4550 (5)	0.5585 (3)	−0.1937 (2)	0.0508 (8)	
H23	−0.4746	0.6280	−0.1976	0.061*	
C22	−0.5228 (4)	0.5035 (2)	−0.2675 (2)	0.0461 (7)	
C23	−0.4960 (5)	0.4015 (3)	−0.2652 (3)	0.0542 (9)	
H24	−0.4279	0.3667	−0.2161	0.065*	
C24	−0.5667 (6)	0.3510 (3)	−0.3331 (3)	0.0628 (10)	
H25	−0.5495	0.2813	−0.3297	0.075*	
C25	−0.6625 (5)	0.3998 (3)	−0.4062 (3)	0.0591 (9)	
H26	−0.7109	0.3643	−0.4532	0.071*	
C26	−0.6874 (5)	0.5011 (3)	−0.4105 (2)	0.0533 (8)	
C27	−0.6184 (5)	0.5528 (3)	−0.3409 (2)	0.0510 (8)	
H27	−0.6372	0.6224	−0.3438	0.061*	
C28	−0.7845 (5)	0.5554 (3)	−0.4867 (3)	0.0626 (10)	
C29	0.2595 (5)	0.2239 (3)	0.2380 (3)	0.0611 (10)	
C30	0.1058 (7)	0.2780 (3)	0.2934 (4)	0.0814 (13)	
F1	0.1449 (15)	0.3562 (8)	0.3268 (9)	0.132 (5)	0.593 (15)
F2	0.0334 (14)	0.2268 (7)	0.3564 (11)	0.156 (6)	0.593 (15)
F3	−0.0247 (13)	0.3127 (8)	0.2334 (8)	0.144 (5)	0.593 (15)
F1'	0.141 (3)	0.2689 (11)	0.3907 (7)	0.145 (6)	0.407 (15)
F2'	−0.0425 (19)	0.2452 (15)	0.2912 (13)	0.153 (7)	0.407 (15)
F3'	0.093 (2)	0.3702 (7)	0.2843 (13)	0.132 (7)	0.407 (15)

### Atomic displacement parameters (Å^2^)

**Table:**

	*U*^11^	*U*^22^	*U*^33^	*U*^12^	*U*^13^	*U*^23^
Ag1	0.0812 (3)	0.0767 (3)	0.0630 (2)	−0.01527 (17)	−0.01994 (17)	−0.03412 (17)
N1	0.0622 (17)	0.0518 (17)	0.0435 (16)	−0.0139 (13)	−0.0157 (13)	−0.0136 (12)
N2	0.0567 (15)	0.0441 (15)	0.0412 (15)	−0.0050 (12)	−0.0149 (12)	−0.0128 (11)
N3	0.0594 (16)	0.0477 (16)	0.0452 (16)	−0.0078 (13)	−0.0152 (13)	−0.0137 (12)
N4	0.079 (2)	0.103 (3)	0.050 (2)	−0.011 (2)	−0.0193 (18)	−0.0021 (19)
N5	0.0699 (18)	0.0492 (18)	0.0460 (16)	−0.0064 (14)	−0.0174 (14)	−0.0158 (13)
N6	0.0672 (17)	0.0418 (15)	0.0435 (15)	−0.0091 (13)	−0.0175 (13)	−0.0097 (12)
N7	0.0606 (16)	0.0450 (15)	0.0390 (15)	−0.0093 (12)	−0.0137 (13)	−0.0115 (12)
N8	0.103 (3)	0.089 (3)	0.061 (2)	−0.019 (2)	−0.037 (2)	0.003 (2)
O1	0.0821 (17)	0.0408 (14)	0.0686 (17)	−0.0027 (12)	−0.0307 (13)	−0.0155 (11)
O2	0.0886 (18)	0.0389 (14)	0.0550 (15)	−0.0067 (12)	−0.0241 (13)	−0.0092 (10)
O3	0.162 (3)	0.074 (2)	0.0531 (18)	−0.024 (2)	−0.0202 (19)	0.0046 (15)
O4	0.110 (2)	0.078 (2)	0.093 (2)	0.0287 (18)	−0.046 (2)	−0.0184 (17)
C1	0.085 (3)	0.042 (2)	0.064 (2)	−0.0097 (18)	−0.029 (2)	−0.0104 (17)
C2	0.084 (2)	0.0413 (19)	0.051 (2)	−0.0106 (17)	−0.0296 (18)	−0.0026 (15)
C3	0.0457 (16)	0.0413 (17)	0.0390 (16)	−0.0081 (13)	−0.0106 (13)	−0.0088 (13)
C4	0.0597 (19)	0.0429 (18)	0.0431 (18)	−0.0100 (15)	−0.0137 (15)	−0.0017 (14)
C5	0.0581 (19)	0.054 (2)	0.0377 (17)	−0.0124 (16)	−0.0144 (15)	−0.0001 (14)
C6	0.0553 (18)	0.0424 (19)	0.0459 (19)	−0.0102 (15)	−0.0130 (15)	−0.0101 (14)
C7	0.0591 (19)	0.0445 (19)	0.0445 (18)	−0.0062 (15)	−0.0095 (15)	−0.0106 (14)
C8	0.0538 (18)	0.0478 (19)	0.0389 (17)	−0.0095 (14)	−0.0117 (14)	−0.0084 (14)
C9	0.081 (3)	0.045 (2)	0.057 (2)	−0.0056 (17)	−0.0252 (19)	−0.0060 (16)
C10	0.099 (3)	0.047 (2)	0.082 (3)	−0.012 (2)	−0.032 (2)	−0.0144 (19)
C11	0.081 (3)	0.061 (3)	0.065 (3)	−0.016 (2)	−0.023 (2)	−0.0222 (19)
C12	0.058 (2)	0.066 (2)	0.0423 (19)	−0.0110 (17)	−0.0096 (16)	−0.0090 (16)
C13	0.0570 (19)	0.0456 (19)	0.0430 (18)	−0.0064 (15)	−0.0093 (15)	−0.0058 (14)
C14	0.062 (2)	0.076 (3)	0.046 (2)	−0.0118 (19)	−0.0089 (18)	−0.0125 (18)
C15	0.106 (3)	0.041 (2)	0.066 (2)	−0.008 (2)	−0.035 (2)	−0.0093 (17)
C16	0.087 (3)	0.0405 (19)	0.051 (2)	−0.0024 (17)	−0.0313 (19)	−0.0053 (15)
C17	0.0482 (17)	0.0408 (17)	0.0362 (16)	−0.0028 (13)	−0.0035 (13)	−0.0059 (13)
C18	0.068 (2)	0.0406 (18)	0.0413 (18)	0.0011 (15)	−0.0147 (15)	−0.0067 (14)
C19	0.062 (2)	0.056 (2)	0.0422 (18)	−0.0019 (16)	−0.0168 (15)	−0.0095 (15)
C20	0.0528 (18)	0.0416 (19)	0.0394 (17)	−0.0034 (14)	−0.0096 (14)	−0.0105 (13)
C21	0.064 (2)	0.0436 (19)	0.047 (2)	−0.0095 (15)	−0.0115 (16)	−0.0071 (15)
C22	0.0551 (18)	0.0457 (19)	0.0403 (17)	−0.0137 (15)	−0.0080 (14)	−0.0072 (14)
C23	0.065 (2)	0.046 (2)	0.054 (2)	−0.0105 (16)	−0.0137 (17)	−0.0035 (15)
C24	0.077 (2)	0.047 (2)	0.068 (3)	−0.0150 (18)	−0.013 (2)	−0.0151 (17)
C25	0.071 (2)	0.057 (2)	0.054 (2)	−0.0169 (18)	−0.0123 (18)	−0.0179 (17)
C26	0.059 (2)	0.061 (2)	0.0434 (19)	−0.0170 (17)	−0.0117 (15)	−0.0073 (16)
C27	0.064 (2)	0.0462 (19)	0.0454 (19)	−0.0128 (16)	−0.0148 (16)	−0.0039 (14)
C28	0.077 (2)	0.064 (2)	0.052 (2)	−0.019 (2)	−0.0222 (19)	−0.0061 (18)
C29	0.086 (3)	0.0379 (19)	0.061 (2)	−0.0105 (18)	−0.021 (2)	0.0021 (16)
C30	0.094 (3)	0.052 (3)	0.095 (4)	−0.002 (2)	−0.009 (3)	0.010 (3)
F1	0.149 (7)	0.122 (9)	0.135 (8)	−0.021 (6)	0.023 (6)	−0.076 (7)
F2	0.131 (7)	0.126 (7)	0.189 (11)	0.019 (5)	0.075 (7)	0.075 (8)
F3	0.124 (6)	0.121 (7)	0.180 (8)	0.056 (5)	−0.066 (6)	−0.036 (6)
F1'	0.234 (14)	0.121 (10)	0.067 (5)	0.047 (9)	−0.009 (7)	−0.023 (6)
F2'	0.114 (8)	0.184 (15)	0.171 (13)	−0.061 (9)	0.031 (8)	−0.029 (11)
F3'	0.171 (12)	0.037 (5)	0.168 (13)	0.038 (6)	0.048 (9)	0.032 (7)

### Geometric parameters (Å, °)

**Table:**

Ag1—N5	2.143 (3)	C9—C10	1.369 (5)
Ag1—N1	2.147 (3)	C9—H33	0.9500
N1—C5	1.327 (4)	C10—C11	1.372 (6)
N1—C1	1.338 (5)	C10—H34	0.9500
N2—C6	1.353 (4)	C11—C12	1.381 (6)
N2—N3	1.372 (4)	C11—H35	0.9500
N2—H40	0.8793	C12—C13	1.388 (5)
N3—C7	1.275 (4)	C12—C14	1.440 (6)
N4—C14	1.136 (5)	C13—H36	0.9500
N5—C19	1.330 (5)	C15—C16	1.363 (5)
N5—C15	1.338 (5)	C15—H22	0.9500
N6—C20	1.364 (4)	C16—C17	1.381 (5)
N6—N7	1.367 (4)	C16—H21	0.9500
N6—H39	0.9025	C17—C18	1.383 (4)
N7—C21	1.270 (4)	C17—C20	1.493 (4)
N8—C28	1.135 (5)	C18—C19	1.360 (5)
O1—C6	1.215 (4)	C18—H20	0.9500
O2—C20	1.214 (4)	C19—H19	0.9500
O3—C29	1.216 (4)	C21—C22	1.458 (5)
O4—C29	1.218 (4)	C21—H23	0.9500
C1—C2	1.363 (5)	C22—C27	1.381 (5)
C1—H28	0.9500	C22—C23	1.393 (5)
C2—C3	1.378 (5)	C23—C24	1.369 (5)
C2—H29	0.9500	C23—H24	0.9500
C3—C4	1.381 (4)	C24—C25	1.376 (5)
C3—C6	1.500 (4)	C24—H25	0.9500
C4—C5	1.365 (5)	C25—C26	1.382 (5)
C4—H30	0.9500	C25—H26	0.9500
C5—H31	0.9500	C26—C27	1.391 (5)
C7—C8	1.454 (4)	C26—C28	1.443 (5)
C7—H32	0.9500	C27—H27	0.9500
C8—C13	1.380 (5)	C29—C30	1.526 (7)
C8—C9	1.402 (5)		
			
N5—Ag1—N1	174.20 (11)	C11—C12—C14	120.6 (3)
C5—N1—C1	116.6 (3)	C13—C12—C14	118.9 (3)
C5—N1—Ag1	121.0 (2)	C8—C13—C12	120.1 (3)
C1—N1—Ag1	121.8 (2)	C8—C13—H36	119.9
C6—N2—N3	117.8 (3)	C12—C13—H36	119.9
C6—N2—H40	128.2	N4—C14—C12	179.0 (5)
N3—N2—H40	113.6	N5—C15—C16	123.9 (3)
C7—N3—N2	116.5 (3)	N5—C15—H22	118.0
C19—N5—C15	116.7 (3)	C16—C15—H22	118.0
C19—N5—Ag1	121.9 (2)	C15—C16—C17	119.2 (3)
C15—N5—Ag1	121.2 (2)	C15—C16—H21	120.4
C20—N6—N7	118.7 (3)	C17—C16—H21	120.4
C20—N6—H39	121.2	C16—C17—C18	116.8 (3)
N7—N6—H39	120.1	C16—C17—C20	126.4 (3)
C21—N7—N6	115.9 (3)	C18—C17—C20	116.8 (3)
N1—C1—C2	123.9 (3)	C19—C18—C17	120.6 (3)
N1—C1—H28	118.0	C19—C18—H20	119.7
C2—C1—H28	118.0	C17—C18—H20	119.7
C1—C2—C3	119.0 (3)	N5—C19—C18	122.8 (3)
C1—C2—H29	120.5	N5—C19—H19	118.6
C3—C2—H29	120.5	C18—C19—H19	118.6
C2—C3—C4	117.5 (3)	O2—C20—N6	123.2 (3)
C2—C3—C6	125.2 (3)	O2—C20—C17	120.8 (3)
C4—C3—C6	117.3 (3)	N6—C20—C17	115.9 (3)
C5—C4—C3	119.7 (3)	N7—C21—C22	121.1 (3)
C5—C4—H30	120.1	N7—C21—H23	119.5
C3—C4—H30	120.1	C22—C21—H23	119.5
N1—C5—C4	123.3 (3)	C27—C22—C23	118.7 (3)
N1—C5—H31	118.4	C27—C22—C21	119.8 (3)
C4—C5—H31	118.4	C23—C22—C21	121.5 (3)
O1—C6—N2	124.1 (3)	C24—C23—C22	120.7 (3)
O1—C6—C3	120.2 (3)	C24—C23—H24	119.6
N2—C6—C3	115.6 (3)	C22—C23—H24	119.6
N3—C7—C8	119.3 (3)	C23—C24—C25	120.8 (3)
N3—C7—H32	120.4	C23—C24—H25	119.6
C8—C7—H32	120.4	C25—C24—H25	119.6
C13—C8—C9	118.5 (3)	C24—C25—C26	119.3 (3)
C13—C8—C7	120.5 (3)	C24—C25—H26	120.4
C9—C8—C7	121.0 (3)	C26—C25—H26	120.4
C10—C9—C8	121.0 (3)	C25—C26—C27	120.2 (3)
C10—C9—H33	119.5	C25—C26—C28	121.2 (3)
C8—C9—H33	119.5	C27—C26—C28	118.6 (3)
C9—C10—C11	120.1 (4)	C22—C27—C26	120.3 (3)
C9—C10—H34	119.9	C22—C27—H27	119.8
C11—C10—H34	119.9	C26—C27—H27	119.8
C10—C11—C12	119.8 (3)	N8—C28—C26	178.3 (4)
C10—C11—H35	120.1	O3—C29—O4	131.0 (4)
C12—C11—H35	120.1	O3—C29—C30	115.8 (4)
C11—C12—C13	120.5 (3)	O4—C29—C30	113.3 (4)
			
N5—Ag1—N1—C5	122.8 (10)	C14—C12—C13—C8	−179.2 (3)
N5—Ag1—N1—C1	−66.4 (11)	C11—C12—C14—N4	166 (27)
C6—N2—N3—C7	177.3 (3)	C13—C12—C14—N4	−15 (28)
N1—Ag1—N5—C19	56.5 (11)	C19—N5—C15—C16	−1.2 (7)
N1—Ag1—N5—C15	−127.5 (10)	Ag1—N5—C15—C16	−177.4 (4)
C20—N6—N7—C21	175.5 (3)	N5—C15—C16—C17	1.6 (7)
C5—N1—C1—C2	1.0 (6)	C15—C16—C17—C18	−0.9 (6)
Ag1—N1—C1—C2	−170.2 (3)	C15—C16—C17—C20	178.6 (4)
N1—C1—C2—C3	−0.1 (7)	C16—C17—C18—C19	−0.1 (5)
C1—C2—C3—C4	−0.8 (6)	C20—C17—C18—C19	−179.6 (3)
C1—C2—C3—C6	177.1 (4)	C15—N5—C19—C18	0.1 (5)
C2—C3—C4—C5	0.9 (5)	Ag1—N5—C19—C18	176.3 (3)
C6—C3—C4—C5	−177.2 (3)	C17—C18—C19—N5	0.5 (6)
C1—N1—C5—C4	−0.9 (5)	N7—N6—C20—O2	−3.4 (5)
Ag1—N1—C5—C4	170.4 (3)	N7—N6—C20—C17	178.2 (3)
C3—C4—C5—N1	0.0 (5)	C16—C17—C20—O2	−174.3 (4)
N3—N2—C6—O1	−2.0 (5)	C18—C17—C20—O2	5.1 (5)
N3—N2—C6—C3	177.5 (3)	C16—C17—C20—N6	4.1 (5)
C2—C3—C6—O1	−160.7 (4)	C18—C17—C20—N6	−176.4 (3)
C4—C3—C6—O1	17.1 (5)	N6—N7—C21—C22	178.3 (3)
C2—C3—C6—N2	19.7 (5)	N7—C21—C22—C27	178.4 (3)
C4—C3—C6—N2	−162.4 (3)	N7—C21—C22—C23	−0.6 (5)
N2—N3—C7—C8	177.6 (3)	C27—C22—C23—C24	−1.6 (5)
N3—C7—C8—C13	174.7 (3)	C21—C22—C23—C24	177.4 (3)
N3—C7—C8—C9	−5.2 (5)	C22—C23—C24—C25	1.5 (6)
C13—C8—C9—C10	1.2 (6)	C23—C24—C25—C26	−0.2 (6)
C7—C8—C9—C10	−178.8 (4)	C24—C25—C26—C27	−0.9 (6)
C8—C9—C10—C11	−0.1 (7)	C24—C25—C26—C28	179.1 (4)
C9—C10—C11—C12	−1.1 (7)	C23—C22—C27—C26	0.5 (5)
C10—C11—C12—C13	1.2 (6)	C21—C22—C27—C26	−178.6 (3)
C10—C11—C12—C14	−179.6 (4)	C25—C26—C27—C22	0.8 (5)
C9—C8—C13—C12	−1.1 (5)	C28—C26—C27—C22	−179.2 (3)
C7—C8—C13—C12	178.9 (3)	C25—C26—C28—N8	−134 (17)
C11—C12—C13—C8	−0.1 (6)	C27—C26—C28—N8	46 (17)

### Hydrogen-bond geometry (Å, °)

**Table:**

*D*—H···*A*	*D*—H	H···*A*	*D*···*A*	*D*—H···*A*
N2—H40···O4^i^	0.88	1.93	2.805 (4)	172
N6—H39···O3^ii^	0.90	2.13	2.936 (4)	149

Symmetry codes: (i) −*x*+1, −*y*+1, −*z*+1; (ii) −*x*, −*y*+1, −*z*.
